# Nanoengineered Interfaces, Coatings, and Structures by Plasma Techniques

**DOI:** 10.3390/nano7120449

**Published:** 2017-12-15

**Authors:** Krasimir Vasilev, Melanie Macgregor Ramiasa

**Affiliations:** School of Engineering, Future Industries Institute, University of South Australia, Adelaide, SA 5095, Australia; melanie.ramiasa@unisa.edu.au

Nanoparticles, nanotubes, nanobelts, nanoneedles, nanosheets, nanowires, nanopillars: the variety of nanostructured interfaces that can be created and modified using plasma processes is virtually endless. This is great news for modern technologies because plasma-generated nanomaterials have unique properties that can benefit many industrial fields, ranging from electronics and photonics to medicine and biology. Using versatile plasma techniques to produce nanoengineered structures has many advantages over other conventional methods. This stems from the fact that plasma processes are environmentally friendly and time-efficient, capable of creating unique materials unachievable by other means. Plasma processes are also readily scalable and transferable to industrial use. In recent years, proactive approaches in this field have led to breakthroughs in plasma nanofabrication, generating nanoengineered materials with properties that could not be equaled via other techniques such as self-assembly or classic lithography.

This Special Issue showcases some of the latest advances in the field of plasma nanoengineering of interfaces, coatings, and structures. High-quality contributions were chosen for their ability to unravel some of the fundamental physical, chemical, and physicochemical mechanisms governing plasma nanostructuring, with the aim to highlight potential applications in various fields, ongoing challenges, and future research directions. As such, this Special Issue gives us the opportunity to reflect on how our field has evolved in the recent years. In the 10 original research papers compiled here, the reader is provided with new insight on how plasmas temper with the properties of nano-objects and how plasmas can be tailored to engineer new nanostructures for targeted technologies.

The fundamental work conducted by Cheng et al. provides valuable insight on the effect of high-energy ion collision on nanostructures [1]. In this case, the nano-objects in question were gold nanowires. The authors demonstrated that ion irradiation strongly modified not only the surface of the gold nanowires with the formation of craters, but also their bulk with the appearance of faults in the crystal structures. This work highlights that, while the beneficial effect of ion irradiation can be used to form organized nanostructures through plasma–wall interactions with the substrate, damages to nanomaterials may also occurs. These plasma-induced modifications of nanostructures should not be underestimated, especially when working with nanoelectronic devices in an irradiation environment. The destructive effect of high-density plasma was also investigated by Lin et al. [2]. The authors tested a new generation of protective coating for silicon-based ceramic used in the semiconductor industry. By studying the erosion behavior of YF_3_ and Y_2_O_3_ coatings under the action of fluorinated plasma, Lin et al. demonstrated that YF_3_ is more robust than Y_2_O_3_ against CF_4_/O_2_ plasma irradiation.

Several articles in this Special Issue have focused on using existing methods or developing new plasma approaches to generate nanostructures and nanotextured interfaces. For instance, in a systematic study, Szymanski et al. developed ways to improve carbon nanotube synthesis technology using microwave atmospheric plasma [3]. Raniszewski et al. also investigated ways to increase the yield of carbon nanotube synthesis, this time via an arc-discharge plasma method with controlled plasma jet temperature [4]. Baquedano et al. optimized a reactive ion etching process and combined it with soft lithography to generate nanobelts and nanopillars on large silicon wafers areas. The structures were produced without using a second layer of photoresist, and featured lateral dimensions below 200 nm and aspect ratios >1.5 [5]. Such nanostructures are the basis of multifunctional devices used in photonics, sensing, and biotechnology. However, their commercial implementation is often held back because of nanofabrication scalability limitations. The work of Baquedano et al. demonstrated that challenges in nanopatterning of large surface areas can be overcome by plasma-assisted nanofabrication approaches. 

Prasad et al. worked with another plasma-enabled method for the production of nanowalls from waste materials [6]. More specifically, a fast plasma-assisted chemical vapor deposition process was used to produce high value-added graphene nanowalls from bagasse, a low-value by-product from the sugarcane industry. They demonstrated that the sustainable nanostructure made from bagasse had comparable or better antifouling properties than copper, depending on the microorganism investigated. As the range of application for plasma-derived coatings in the biomedical field continue to increase, evaluating the antifouling properties of such interfaces is essential. Al-Jumaili et al. assessed the antibacterial activity of nanothin plasma polymer films deposited from geranium essential oil [7]. The plasma polymer of this natural antimicrobial, when deposited at low ignition power, did reduce biofilm formation. Another article in this Special Issue is particularly focused on the applications of plasma-derived, nanoengineered surfaces in implantology. Radtke et al. deposited silver nanoparticles on titanium nanotubes and nanoneedles via plasma-enhanced atomic layer deposition in order to achieve both biointegration and antimicrobial activity for what could become a new generation of nanoengineered implant coatings [8].

Another important field of applied science currently benefitting from novel nanostructured materials is photonics, an area that encompasses all technologies used to generate and harvest light, whose unit is the photon. Bazaka and Jacob investigated the electrical and dielectric properties of plasma polymer thin films for applications in optoelectronic [9]. They used a natural precursor oil and iodine as a dopant to increase the conductivity of the organic film, thus highlighting their potential for use in flexible organic electronic circuits. Baquedano et al. reported on plasma-assisted nanostructuration of solar glass with the aim to improve solar cell efficiency via wettability control and enhanced light transmission [10]. In this work, surface nanoengineering could either increase or decrease the glass hydrophobicity depending on the nanoscale ordering of the surface topography. This work demonstrates how plasma approaches can be particularly useful to achieve self-cleaning and antifogging properties from nanoscale topography, as recently reviewed [11].

In summary, this Special Issue of Nanomaterials entitled "Nanoengineered Interfaces, Coatings, and Structures by Plasma Techniques” compiles a series of research articles demonstrating the potential of plasma techniques for the generation of coatings and interfaces with controlled nanoscaled topography, as well as key, fundamental work underpinning some of the complex mechanisms involved in plasma-based nanoengineering.
